# Wisdom in surgery in the era of artificial intelligence

**DOI:** 10.1308/rcsann.2026.0126

**Published:** 2026-11-01

**Authors:** K Mahawar

**Affiliations:** *Annals* Editor-in-Chief

**Figure:**
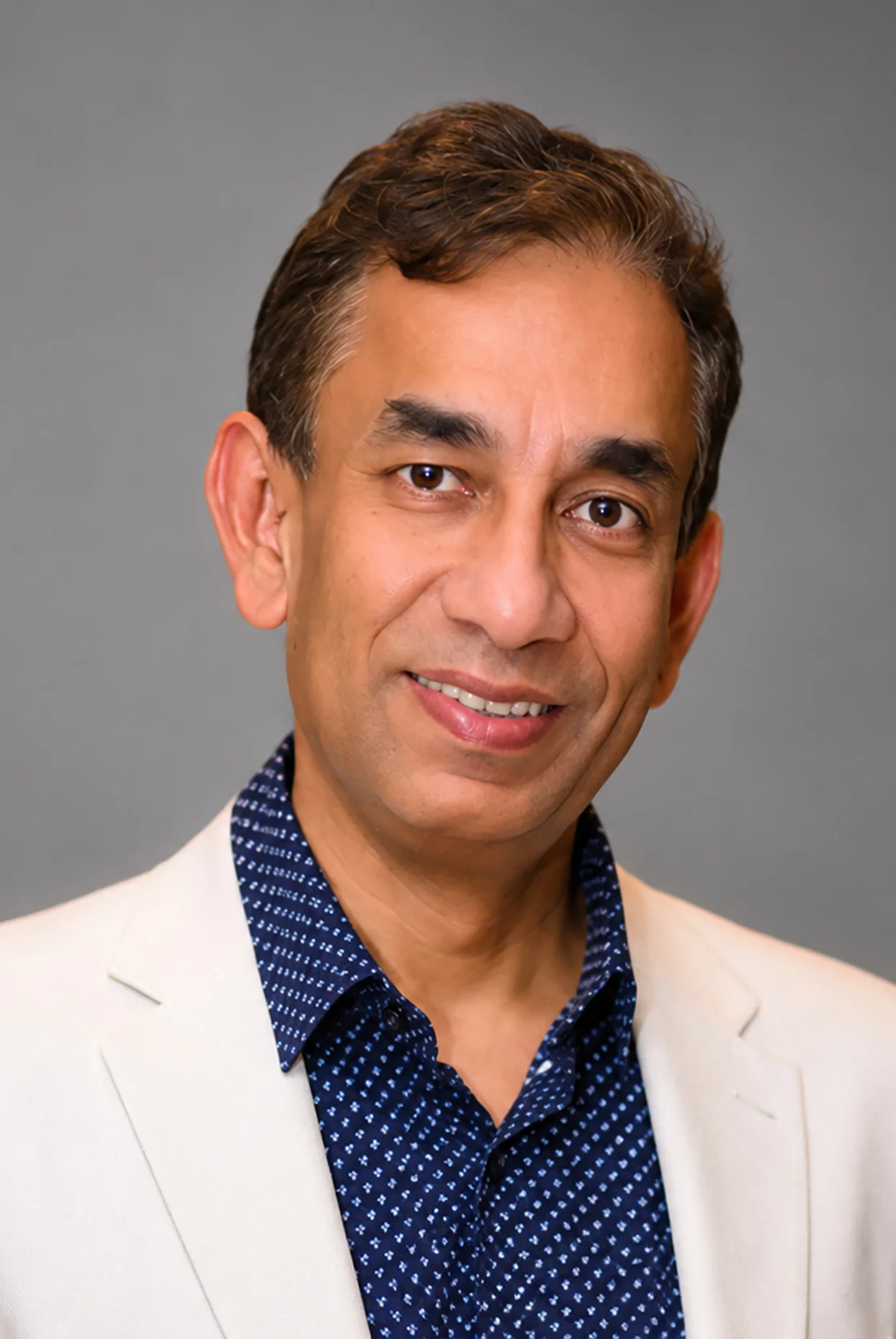


In this issue of the *Annals*, we have several articles on the use of artificial intelligence (AI) in different areas of surgery.^1–3^ Some of the challenges we face in safely incorporating AI into clinical practice are reminiscent of those that ‘my generation’ encountered with the advent of search engines such as Google^®^ approximately a quarter of a century ago. Information became widely available, but wisdom, the ability to distinguish the useful from the useless or even harmful, remained scarce. One of the additional tasks that we clinicians then had to deal with was not only to give patients our views on the illness with which they presented, but also to explain what they had found on the internet. Sometimes that information was useful; more often, it was not.

The ability to delegate tasks to machines, mechanical or electronic, is not new to us humans. We have been on that journey for millennia. What makes AI particularly interesting is that, for the first time, we appear able to outsource some aspects of thinking: to distil a large body of information into a clear, unambiguous output. We may pose a very difficult question and, within seconds, a model will return a confident and compelling answer.

Our questions and problems do not always have a clear solution. There may be hundreds of options from which to choose before deciding on the right one. Such decisions usually require considerable reflection and, dare I say, wisdom. That wisdom usually comes from experience, often painful. What will happen to wisdom in an era of readily available intelligence? Will it become irrelevant, or will it become a scarcer commodity that the world values even more?

Surgeons understand the value of wisdom. That is why even a highly cited randomised controlled trial showing superiority for a defined endpoint often fails to influence practice. Surgeons know that clinical decision making cannot be reduced to a single paper, even if it reports a very high-quality study. We look at a problem from multiple angles: we analyse what the evidence shows and, more importantly, what it does not; discuss it with senior and junior colleagues; attend conferences; consider what our patients are actually experiencing and will find acceptable; and assess what is reproducible in our own practice and hands before making up our minds. That process takes time and is not something any model can easily replicate by scouring global databases in seconds before returning a verdict.

There is another aspect to this. Most clinical journeys involve a series of decisions, and our education and training focus on enabling us to guide patients through that journey from beginning to end and beyond. Although we may be able to outsource some of these steps to AI in the future, it is extremely unlikely that any patient will want every step to be delivered by a model, however intelligent.

It is possible that AI will eventually evolve into something capable of delivering compassionate, wise, patient-centred care suited to our clinical environment, and of taking responsibility for it. But that day is not near and, if we clinicians remain focused on the core values of ‘good medical (or surgical) practice’, it may never come. We must, however, try to use this technology to provide better care and extend it to previously underserved populations, just as we have done with thousands of other technological advances since the days of Hippocrates and Sushruta.
